# Chain length‐dependent inulin alleviates diet‐induced obesity and metabolic disorders in mice

**DOI:** 10.1002/fsn3.2283

**Published:** 2021-05-07

**Authors:** Liangkui Li, Lu Zhang, Linkang Zhou, Meijun Jin, Li Xu

**Affiliations:** ^1^ State Key Laboratory of Membrane Biology and Tsinghua‐Peking Center for Life Sciences School of Life Sciences Tsinghua University Beijing China

**Keywords:** gut microbiota, insulin sensitivity, inulin, obesity

## Abstract

Dietary fiber is regarded to improve host metabolic disorders through modulating gut microbiota. The study was to investigate the effects of inulin with different degree of polymerization (DP) on adiposity, related metabolic syndrome, and the possible mechanisms from the points of gut microbiota and metabolite changes. C57Bl/6J male mice were randomly allocated to normal diet (ND) group, high‐fat diet (HFD) group, two HFD groups with short‐chain inulin (HFD‐S) and medium and long‐chain inulin (HFD‐ML) for 8 weeks. Compared with HFD treatment, ML‐inulin supplementation significantly decreased weight gain, hepatic steatosis, chronic inflammation, and increased insulin sensitivity, energy expenditure and thermogenesis. This could be mimicked by S‐inulin supplementation to some degree although it is not as effective as ML inulin. Also, mice treated with S and ML inulin had a remarkable alternation in the composition of gut microbiota and increased the production of short‐chain fatty acids (SCFAs). However, reduced serum levels of essential fatty acids, vitamins B1 and B3 by HFD were further decreased by both inulin supplementations. ML inulin can prevent HFD‐induced obesity and the associated metabolic disorders, and may be used as novel gut microbiota modulator to prevent HFD‐induced gut dysbiosis and metabolic disorders.

## Graphical abstract

*Pogostemon cablin* extract contained potential anticancer compound to suppress HL‐60 cell growth by blocking cell cycle progression and inducing cell apoptosis.

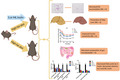

## 1 INTRODUCTION

Obesity and its related metabolic diseases, including cardiovascular disease and diabetes mellitus, are among humanity's most widespread public health problems today (World Health Organization, 2018). Increasing evidences have demonstrated that altered host‐microbiota interaction in the gut contributes to the development of obesity (Ley et al., 2005; Turnbaugh et al., 2009; Vrieze et al., 2012; Zhao, 2013). Therefore, modulating gut microbiota may be potential targets for the prevention and treatment of obesity.

Society changes in dietary habits, especially considerably lower dietary fiber intake than recommended level of 25 g/d, are considered to affect gut microbiota, and thereby increasing incidence of obesity and other metabolic disorders (Galas et al., 2013; Reynolds et al., 2019; Sonnenburg and Sonnenburg, 2014). Inulin, as a fermentable dietary fiber, are extracted from chicory root and linked by a β(2–1) fructofuranosyl bond. Inulin is broadly used in dietary addition to improve human health, including protecting against weight gain, improving insulin sensitivity and associated parameters of metabolic syndrome (Aliasgharzadeh et al., 2015; Genta et al., 2009; Russo et al., 2010). It is noteworthy that the fermentation rate and products of inulin may be impacted by chemistry or DP, as any fermentable carbohydrates must be hydrolyzed to monosaccharides prior to fermentation by bacteria (Campbell et al., 1997; Nilsson & Nyman, 2005). This differential fermentation profile results in DP‐dependent effects (Chen et al., 2017; He et al., 2017). One of previous studies demonstrated that long‐chain inulin but not short‐chain inulin attenuated the incidence of type 1 diabetes (Chen et al., 2017). However, relatively little information is available regarding the comparative effects of inulin with different DP on gut microbiota and physiological responses; moreover, systematical details are limited even in animal models. In addition, the understanding on molecular mechanism of fiber supplementation in improving metabolic syndrome is still uncovered (Aleixandre & Miguel, 2008; Canfora Emanuel et al., 2019). Broadly, gut metabolite profiles are jointly controlled by diets, gut microbiota and host, which is regarded as a predominant contribution to host health status (Dorrestein et al., 2014). However, very few studies have been conducted using metabolomics to explore the biological effects of inulin administration on the host.

In this work, we systematically examined and compared the effects of inulin with different DP on adiposity and its associated parameters of metabolic syndrome. We used different indexes, including body weight, tissue weight, relative lipid contents in fat pad and liver, energy expenditure and insulin sensitivity, to judge mouse performance and demonstrated ML inulin is better than S inulin. Interestingly, we found that HFD‐induced decreases in essential fatty acids and vitamin B were further decreased by inulin supplementation, indicating the necessity of their supplementation for those people under such diets.

## 2 MATERIALS AND METHODS

### 2.1 Animals and diets

Mouse experiments were performed in the animal facility of Tsinghua University (Beijing, China). All animal experiments were approved by the Institutional Animal Care and Use Committee of Tsinghua University. C57Bl/6J male mice aged six weeks (Laboratory Animal Research Center, Tsinghua University) were individually housed in a controlled environment (22℃, 12‐hr light/dark cycle) with free access to food and water. After two weeks of acclimatization on a normal chow diet (ND), mice were randomly divided into four groups (*n* = 8), including ND, high‐fat diet (HFD, 60% kcal% fat), and two HFD groups with 10% (w/w) short‐chain inulin (HFD‐S), and the mixture of medium and long‐chain inulin (HFD‐ML) added to the HFD. The major composition of HFD was shown in Supplementary Table S1. The total calories of HFD, HFD‐S and HFD‐ML per gram were similar among them (Supplementary Table S2). Short‐chain inulin (DP: 2–9) and medium and long inulin (DP: 17–24) were kindly offered by Wuhan Inuling Bio_Tech Co., Ltd in the present study. Body weight gain and food intake of animals were measured weekly. Fecal samples were collected for microbial analysis at the end of the eighth week.

### 2.2 Serum biochemical analysis

The concentrations of serum non‐esterified fatty acids (NEFAs) and triglycerides (TAG) were determined using a serum triglyceride determination kit (Sigma) and Lab Assay NEFA kit (Wako Pure Chemical Industries, Japan) following the manufacturer's instructions, respectively. The concentration of serum cholesterol level was measured by using cholesterol quantification kit (MAK043, Sigma, USA). The serum concentrations of mouse interleukin‐6 (IL‐6) and tumor necrosis factor‐α (TNF‐α) were determined using the Mouse IL‐6 ELISA Ready‐SETGO Kits and Mouse TNF‐αELISA Ready‐SETGO Kits (eBioseience, USA). Serum concentration of insulin was determined using Ultra Sensitive Mouse Insulin ELISA Kit (Crystal Chem, USA).

### 2.3 Mouse metabolic studies

At week 8, fat and lean body masses were measured by ^1^H minispec system (LF90II, Bruker Optik, Germany) in mice. In addition, using PhenoMaster/LabMaster System (TSE Systems GmbH, Bad Homburg, Germany), four mice randomly chosen from each group were individually placed in metabolic cages to measure O_2_ consumption and the respiratory exchange rate. Mice were monitored for 96 hr, and data were collected at intervals of 27 min after a 2‐days adaptation period. Parameters were compared between HFD and HFD‐S or HFD‐ML without considering body weight differences.

### 2.4 OGTTs and ITTs

Blood glucose concentrations were measured through tail veil bleeding with glucose analyzer (GM9, Analox Instruments Ltd, UK). Mice were gavaged with 1 g glucose per kg body weight to overnight‐fasted mice as oral glucose tolerance tests (OGTTs). After fasting 6 hr, an intraperitoneal injection of insulin (1 units per kg body weight) was administered for insulin tolerance tests (ITTs).

### 2.5 RT‐PCR

Total RNA was isolated from white adipose tissue (WAT) and liver of mice using TRIzol (Thermo Fisher, USA) in accordance with the manufacturer's instructions. First strand cDNA synthesis was conducted with RevertAid First Strand cDNA Synthesis Kit (Thermo Scientific). RT‐PCR detection of gene expression levels were analyzed using the Power SYBR Green PCR Master Mix (Applied Biosystems) on an ABI 7,500 (Applied Biosystems) with reaction volumes of 20 ml. Beta‐actin gene was used as reference gene. The primer sequences used are listed in Supplementary Table S3.

### 2.6 Histological analysis

WAT and livers from mice were excised and fixed in 10% formalin buffer, dehydrated, embedded in paraffin blocks and sections at 5 μm. The sections were then stained with hematoxylin and eosin (H&E staining).

### 2.7 Tissue lipid content

Isolation and measurement of total lipid from mouse tissues have been previously described (Zhou et al., 2018). Briefly, dried lipids were reconstituted in chloroform/ methanol 2:1 and loaded onto a thin‐layer chromatography plate (Sigma, USA). The lipids were resolved in a hexane/diethyl ether/acetic acid (70:30:1, v/v) solution. The thin‐layer chromatography plate was sprayed with 10% CuSO4 in 10% phosphoric acid and were developed by drying in an oven at 120℃.

### 2.8 Gut microbiota analysis

Genomic DNA was extracted by the PowerSoil NDA Isolation Kit (12888–50, MOBIO, USA) according to the manufacturer's instructions. Nano Drop (Thermo Scientific ^TM^) was applied to determine the DNA concentration, roughly reflecting the bacterial amounts in feces. The total DNA was diluted to 1 ng/μl using sterile water and stored at −80℃ until it was measured by Novogene (Beijing, China), and the isolation was confirmed by 1% agarose gels. The V4 hypervariable region of 16S rDNA gene was amplified with specific primers (515 F and 806R). Sequencing libraries were generated using Ion Plus Fragment Library Kit 48 rxns (Thermo Scientific) following manufacturer's recommendations. The quality of library was assessed on the Qubit@ 2.0 Fluorometer (Thermo Scientific). Finally, the library was sequenced on an Ion S5TM XL platform and 400 bp/600 bp single‐end reads were generated.

Single‐end reads were assigned to samples according to their unique barcode. Sequences were analyzed with Unparsed software (Uparse v7.0.1001). Sequences with ≥97% similarity were assigned to the same operational taxonomic units (OTUs). Alpha diversity of each microbiota community was calculated through six indices, including Observed‐species, Shannon index, Chao1 index (QIIME software, Version 1.7.0). Beta diversity on weighted unifrac were calculated by QIIME software (Version 1.7.0), and Principal Coordinate Analysis (PCoA) was displayed by WGCNA package, stat packages and ggplot2 package in R software (Version 2.15.3).

### 2.9 Analysis of SCFAs

Fecal SCFAs were isolated and measured using the method described by Han and colleagues with minor modifications (Han et al., 2015). Briefly, 100 mg fecal content samples were first homogenized in 1 ml of 55% Acetonitrile/water as the analytical solvent (v/v). After centrifugation at 4,000 g for 10 min, 40 μl supernatant was transferred to a clean Eppendorf tube, and combined 20 μl 200 mM 3NPH‐HCl Acetonitrile/ water and 20 μl 120 mM EDC‐HCL‐6% pyridine solvent was incubated at 40°C for 30 min. The reaction mixtures were centrifuged at 10,000 g for 10 min, and then the supernatant was transferred and evaporated to dryness under nitrogen gas. Dried metabolite samples were stored at −80℃ until analysis.

### 2.10 Statistics

All statistical analyses were performed in GraphPad Prism Version 5 (GraphPad Software). All results are expressed as mean ± *SEM*. Two‐tailed Student's *t* test was used with *p* <.05 considered to be significantly different. P‐values are indicated in each figure as *p <.05, **p <.01, ***p <.001.

## 3 RESULTS

### 3.1 Inulin addition prevented diet‐induced obesity and fatty liver with a better effect of ML inulin than S inulin

**FIGURE 1:**
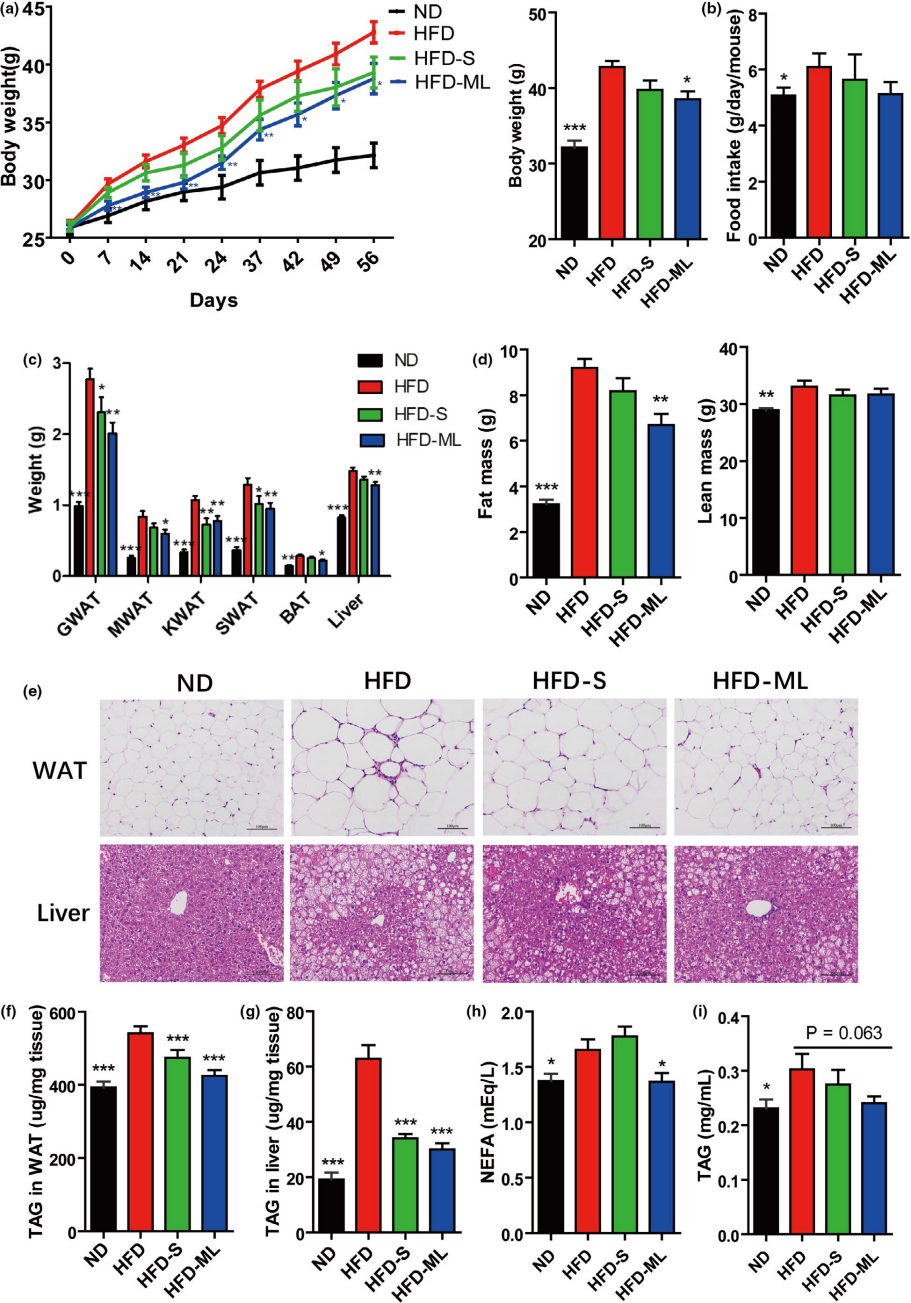
Inulin addition prevents diet‐induced obesity and fatty liver with a better effect of ML Inulin than S inulin. (A) Growth curve reflected by body weight. (B) Food intake. (C) Weights of fat pads and liver. (D) Fat and less masses. (E) Hematoxylin–eosin staining. Scale bars, 100 μm. (F) TAG content in WAT and (G) TAG content in liver. (H) Serum NEFA. (I) Serum TAG. Data represent the mean ± *SEM* from eight mice (*n* = 8). Significance was established using a two‐tailed Student's *t*‐test and all comparisons are relative to HFD group. *p <.05, **p <.01, and ***p <.001

Relative to HFD only, the diet comprised of 10% ML inulin (w/w) prevented diet‐induced weight gain from day 7 onwards, and these findings were not correlated to food intake; also, S inulin had the tendency to prevent diet‐induced obesity (*p* = 0.06) (Figure 1a and 1b). Weights of fat pads from several anatomical locations were significantly decreased in the mice fed with S and ML inulin, while weight of brown adipose tissue (BAT) was only decreased in the mice fed with ML inulin (Figure 1c). The changes of fat pads were also confirmed by MRI analysis with reduced fat mass and similar lean mass (Figure 1d). In addition, LD size and relative level of TAG in WAT were decreased in the mice fed with both S and ML inulin (Figure 1e and 1f). Liver weight was slightly decreased by inulin addition (Figure 1c). Obviously, HFD‐induced hepatic steatosis was effectively prevented by both S and ML inulin treatments, as evidenced by staining liver sections with H&E and decreased TAG accumulation in liver treated by S and ML inulin addition (Figure 1e and 1g). The fasting NEFA levels in serum of ML‐treated mice significantly decreased with a slight decrease in serum TAG level (Figure 1h and 1i). Therefore, inulin addition prevented diet‐induced obesity and fatty liver with a better effect of ML Inulin than S inulin.

### 3.2 Inulin addition increased energy expenditure and mitochondria activity in adipose tissue

**FIGURE 2:**
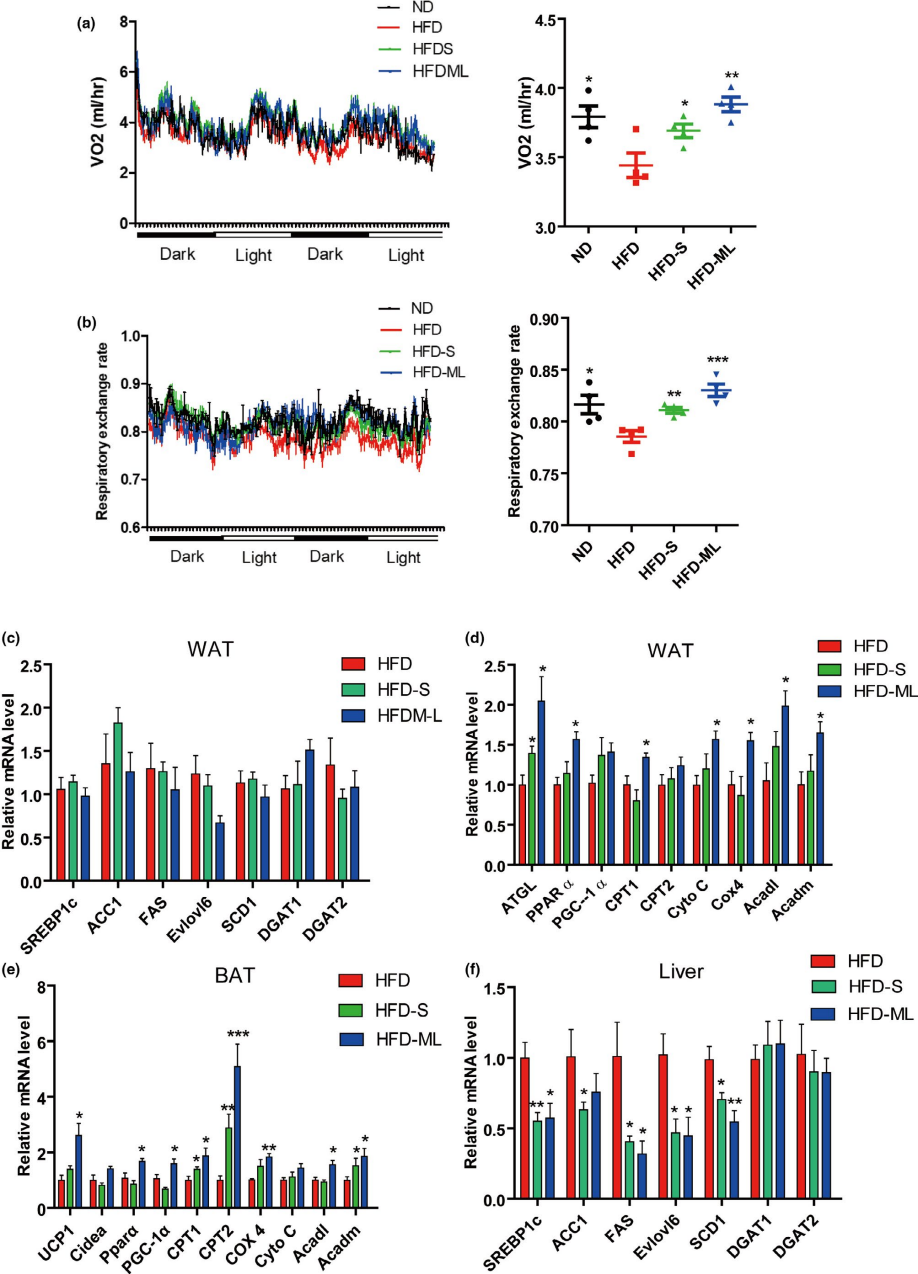
Inulin addition increases energy expenditure and mitochondria activity in adipose tissue. (A) Oxygen consumption (VO2) of mice monitored for 48 hr. (B) Respiratory exchange rate of mice monitored for 48 hr. (C) and (D) Relative mRNA expression levels in WAT. (E) Relative mRNA expression levels in BAT. (F) Relative mRNA expression levels in liver. The data for panels A–B came from four pairs of mice (*n* = 4) and for panels C–F eight pairs of mice (*n* = 8). Data represent the mean ± *SEM*. Significance was established using a two‐tailed Student's *t*‐test and all comparisons are relative to HFD group. *p <.05, **p <.01, and ***p <.001

Next, the metabolic cage experiments were performed using four mice from each group. We observed significantly increased oxygen consumption and respiratory exchange rate in any group of inulin supplementation with a stronger effect in ML‐treatment than that of S‐treatment (Figure 2a and 2b). We measured the mRNA expression of lipogenesis, lipolysis, fatty acids oxidation and thermogenesis genes in WAT and BAT by RT‐PCR. Expression levels of several genes involved in de novo lipogenesis of WAT (ACC1, FAS, Elovl6, SCD1, DGAT1, DGAT2), and their major transcriptional regulator SCRBP1c were not altered in mice treated with S or ML inulin compared with HFD‐fed mice (Figure 2c). In contrast, ATGL, the key gene in the lipolysis, was induced by both S‐ and ML‐treated mice compared with HFD‐fed mice (Figure 2d). Also, we observed a significant upregulation of gene expression related to fatty acid oxidation and oxidative phosphorylation in WAT (PPARα, CPT1, CPT2, Cox4, Cyto C, ACADL, ACADM) in ML‐treated mice (Figure 2d). Concordant with higher BAT activity, the mRNA expression of uncoupling protein 1 (Ucp1), PPARα, PPAR γ coactivator 1a (PGC‐1α), CPT1, CPT2, Cox4, ACADL, ACADM in the BAT of ML‐treated mice were upregulated versus HFD‐treated mice (Figure 2e). The effects of ML inulin treatment were still better than S inulin addition on energy expenditure and related gene expression, consistent with the protective effects against body weight gain (Figure 1). Additionally, the gene expression in lipogenesis was reduced in liver of S‐ or ML‐treated mice compared with that of HFD‐treated mice, including SREBP1c, ACC1, FAS, Elovl6, SCD1 (Figure 2f). This was also consistent with reduced steatosis (Figure 1e and 1g). Therefore, inulin supplementation increased mitochondria activity in fat pads, consequently enhancing energy expenditure and alleviating adiposity and hepatic steatosis.

### 3.3 Inulin addition improved glucose homeostasis and reduced inflammation in diet‐induced obese mice

**FIGURE 3:**
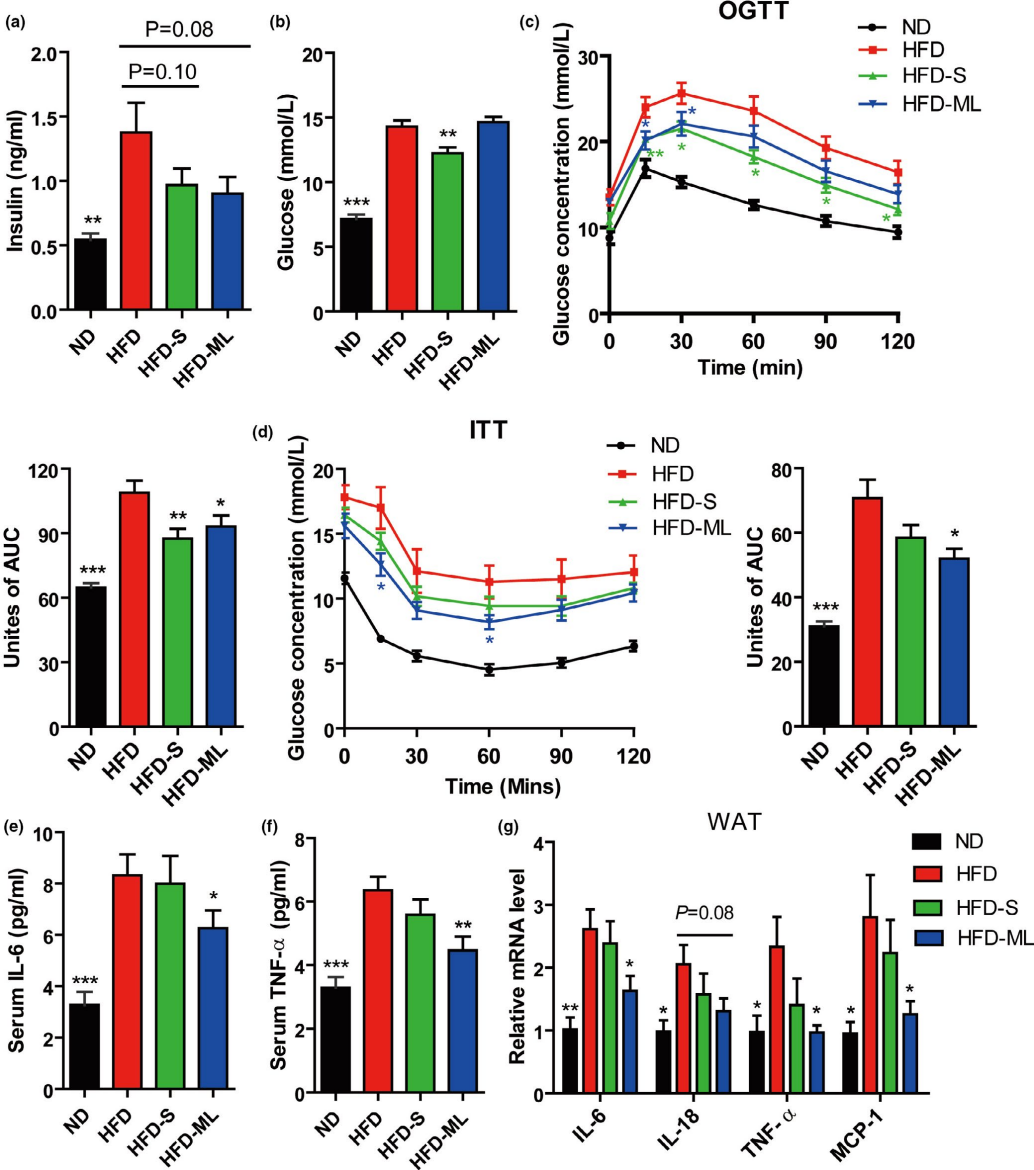
Inulin addition improves glucose homeostasis in diet‐induced obese mice and reduces inflammation. (A) Serum insulin. (B) Serum glucose. (C) OGTTs. (D) ITTs. (E) Serum IL‐6. (F) Serum TNF‐α. (G) Relative mRNA expression levels in the WAT of mice. Data represent the mean ± *SEM* from eight pairs of mice (*n* = 8). Significance was established using a two‐tailed Student's *t*‐test and all comparisons are relative to HFD group. *p <.05, **p <.01, and ***p <.001

In contrast to HFD, both S and ML inulin treatment had a tendency to decrease fasting insulin level in serum (Figure 3a). Blood glucose also significantly decreased in S‐inulin treated mice, which was not observed in ML‐inulin treated mice (Figure 3b). According to oral glucose tolerance tests (OGTTs), both S‐ and ML‐treated mice had significantly lower blood glucose level after the administration of the exogenous load of glucose compared with the HFD‐treated mice as the control (Figure 3c). Insulin tolerance tests (ITTs) demonstrated that ML inulin treatment significantly increased insulin sensitivity, and S‐treated mice had a slightly increased insulin sensitivity (Figure 3d).

Next, inflammation status was detected in these mice. The levels of IL‐6 and TNF‐α in serum from ML‐inulin treated mice were significantly lower than ones in HFD‐treated mice (Figure 3e and 3f). Consistently, the proinflammatory marker genes in WAT, including IL‐6, IL‐18, TNF‐α and monocyte chemotactic protein 1 (MCP‐1) were significantly inhibited (Figure 3g). However, no anti‐inflammatory effect was observed in S inulin treatment. These data supported that protective effects of ML inulin on HFD‐induced obesity and inflammation led to improved glucose homeostasis and insulin sensitivity. S inulin treatment had a minor beneficial effect, not as significant as ML inulin treatment. The markers of inflammation (IL‐6, IL‐18, TNF‐α and MCP‐1), fibrosis (Timp‐1, Collagen‐α1(1) and αSMA) and hepatocellular carcinoma (HCC) (Mmp‐2, Mmp‐3 and Gpc‐3) of liver were not significantly affected by addition of S or ML inulin into HFD compared with that of mice treated with HFD (Figure S1, Supporting Information).

### 3.4 Inulin supplementation alleviates HFD‐induced gut dysbiosis

**FIGURE 4:**
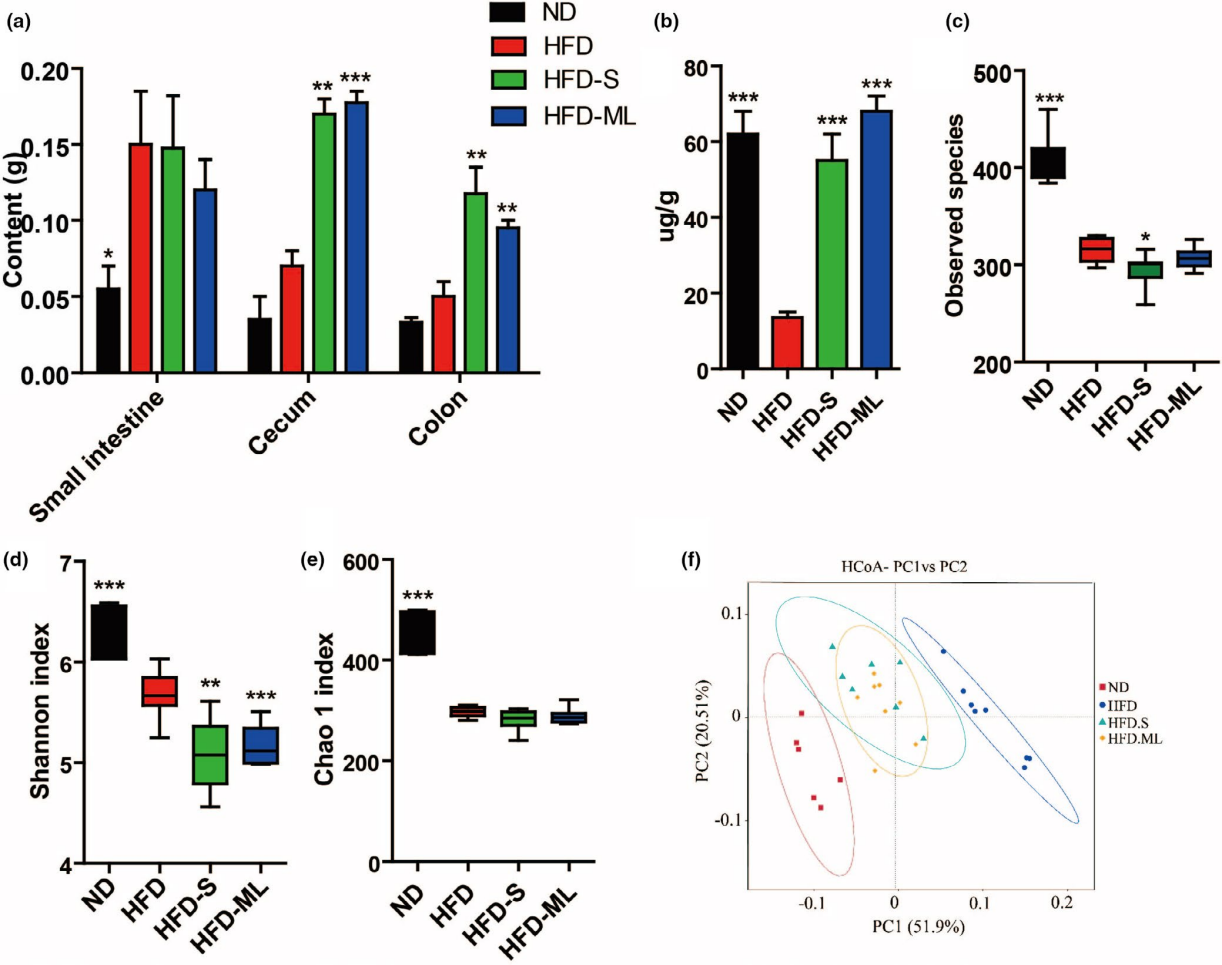
Changes of intestinal content, bacterial DNA amounts and diversity of gut microbiota to inulin in HFD‐fed mice. (A) Content weight of intestinal tract. (B) Total amounts of bacterial DNA in feces. (C) Observed species, (D) Shannon and (E) Chao 1 indexes were used to estimate the alpha‐diversity of the gut microbiota. (F) Beta‐diversity, shown by Weighted UniFrac‐based Principle coordinates analysis (PCoA). Data represent the mean ± *SEM* from eight pairs of mice (*n* = 8). Significance was established using a two‐tailed Student's *t*‐test and all comparisons are relative to HFD group. *p <.05, **p <.01, and ***p <.001

Gut microbiota composition was analyzed by sequencing the V4 region of 16S rDNA in feces after inulin treatments. Content weight in cecum and colon were significantly increased in S‐ and ML‐inulin treated groups in contrast to HFD group (Figure 4a). Compared with ND‐fed mice, HFD treatment resulted in approximately 5‐fold decrease in total amounts of bacterial DNA, indicating HFD‐induced decrease in bacterial amount. Interestingly, HFD‐induced decrease in fecal bacterial DNA amount was fully restored by addition of both S and ML inulin (Figure 4b). Moreover, HFD‐fed mice displayed a low number of observed species and α‐diversity of microbiota evidenced by Shannon and Chao 1 indexes relative to mice fed with ND (Figure 4c‐4e). The number of observed species and Shannon index further dropped in mice fed with inulin supplementation without significant change in Chao 1 index (Figure 4c‐4e). According to Weighted UniFrac‐based PCoA, we observed a distinct clustering of microbial community structure for ND, HFD, and HFD with inulin treatment groups (Figure 4f). However, the microbial community structure from HFD‐S group was similar to that of HFD‐ML group. The groups of inulin additions were located between ND and HFD, indicating that inulin addition partially rescued the gut microbiota changed by HFD to the ND condition.

**FIGURE 5:**
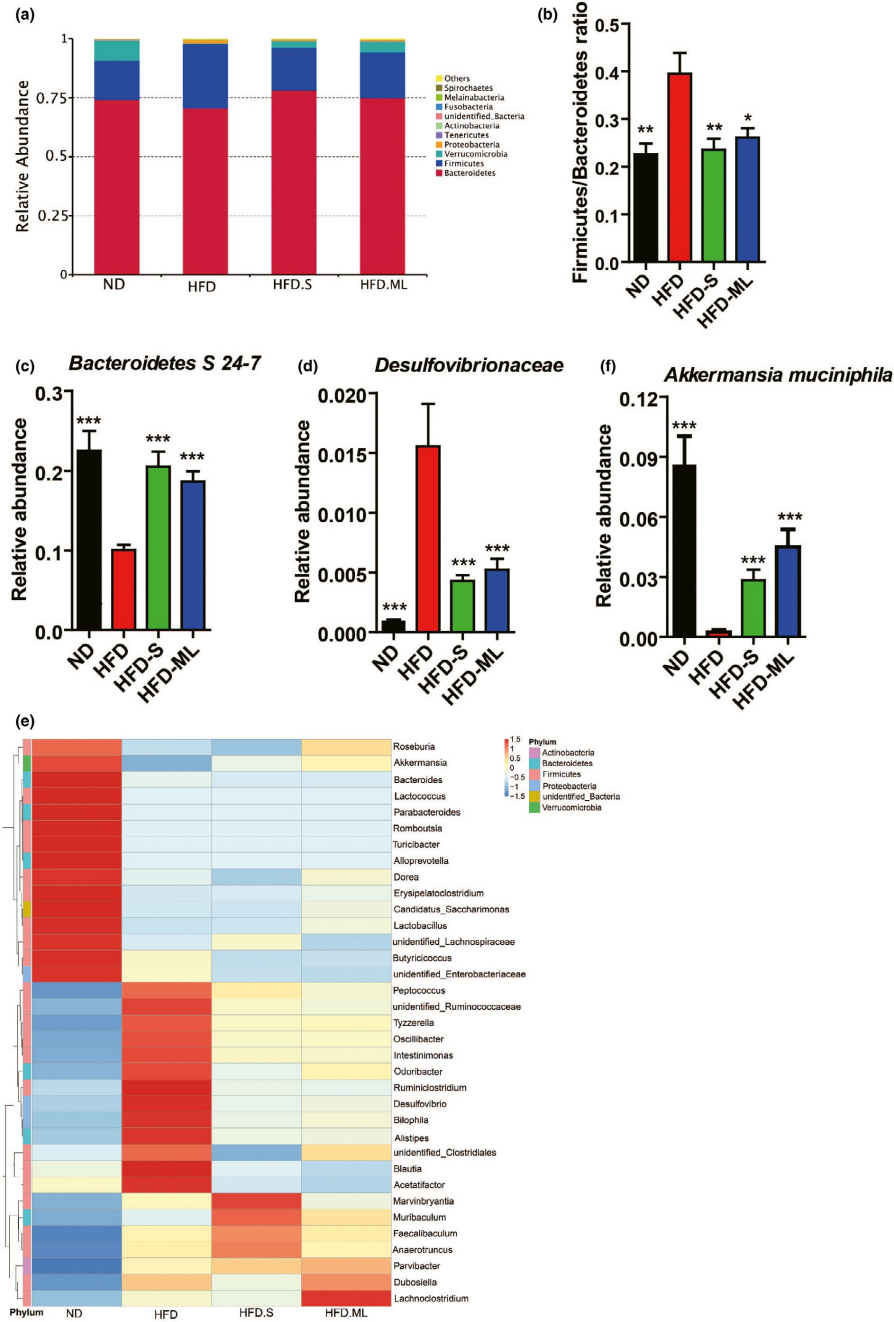
Inulin supplementation alleviates composition of gut microbiota in HFD‐fed mice. (A) Relative abundance of fecal microbiota at phylum level. (B) The ratio of *Firmicutes*/*Bacteroidetes*. (C) Relative abundance of *Bacteroidetes* *S 24–7*. (D) Relative abundance of *Desulfovibrionaceae*. (E) Hierarchically clustered heat map analysis of the top 35 relative abundance of gut microbiota at genus level. (F) Relative abundance of *Akkermansia muciniphila*. Data represent the mean ± *SEM* from eight pairs of mice (*n* = 8). Significance was established using a two‐tailed Student's *t*‐test and all comparisons are relative to HFD group. *p <.05, **p <.01, and ***p <.001

Next, we analyzed the microbiota composition at the phylum level. *Bacteroidetes* and *Firmicutes* are the most abundant phyla in mouse gut (Figure 5a and 5b). The ratio of *Firmicutes*/*Bacteroidetes* was enhanced by HFD in contrast to ND, which was rescued by inulin addition to the similar level to ND condition. Obviously, HFD treatment dramatically resulted in a depletion of *Verrucomicrobia* and appearance of *Proteobacteria*, which was also partly reversed by addition of S or ML inulin into HFD. At the family level, HFD treatment resulted in a substantial decrease in the level of *Bacteroidetes* *S 24–7*, which was fully restored by addition of S or ML inulin, while *Desulfovibrionaceae* experienced an opposite tendency (Figure 5c and 5d). At the genus level, the top 35 changed genera were displayed by heatmap analysis (Figure 5e) Compared to ND, HFD treatment significantly decreased the relative abundances of 15/35 genera, such as *Roseburia* and *AKKermansia*, which could be rescued at some degree by ML‐inulin addition (Figure 5e). In addition, HFD‐treatment significantly increased the relative abundances of *Peptococcus*, *unidentified_Ruminococcaceae*, *Tyzzerella*, *Oscillibacter*, *Intestinimonas*, *Ruminiclostridium*, *unidentified_Clostridiales*, *Blautia*, *Acetatifactor* belonging to *Firmicutes*, and *Odoribacter*, *Alistipes* belonging to *Bacteriodetes*, and *Desulfovibrio*, *Bilophila* belonging to *Proteobacteria*. Interestingly, all of these genera were remarkedly decreased by S or ML addition (Figure 5e). At the species level, HFD treatment caused a depletion in the level of *Akkermansia muciniphila*, which was partly restored by addition of S or ML inulin (Figure 5f).

### 3.5 Changes of fecal SCFAs and serum metabolites by inulin treatment in HFD‐treated mice

**FIGURE 6:**
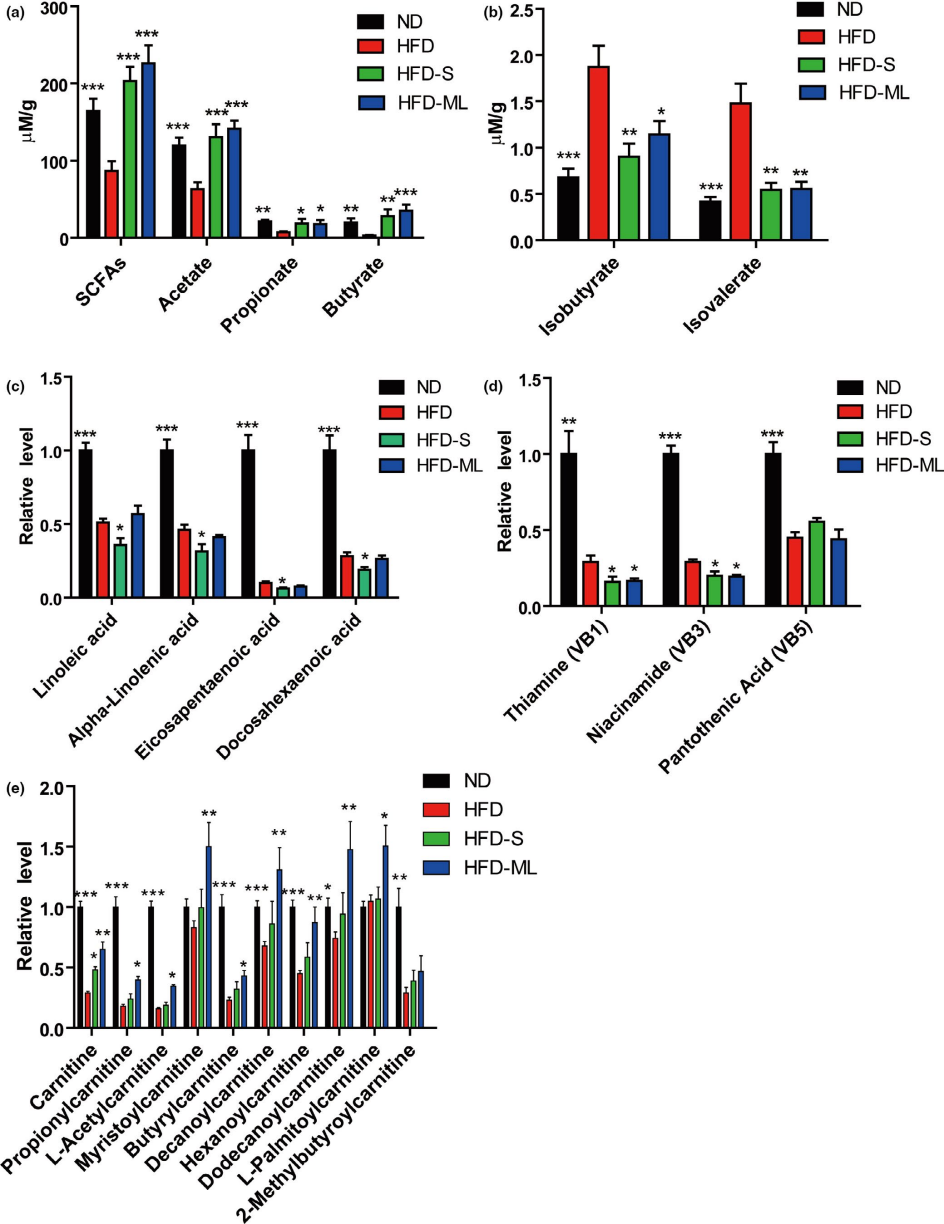
Metabolite changes in fecal and serum by inulin treatment. (A) Content of total SCFAs, acetate, propionate and butyrate in feces. (B) Content of isobutyrate and isovalerate in feces. (C)–(E) Relative level of PUFAs, Vitamins B and Carnitines in serum analyzed by metabolomics, respectively. Data represent the mean ± *SEM* from eight pairs of mice (*n* = 8). Significance was established using a two‐tailed Student's *t*‐test and all comparisons are relative to HFD group. *p <.05, **p <.01, and ***p <.001

Metabolites derived from gut microbiota or the interaction between microbiota and host are the biotransformation of dietary components to host. Therefore, short‐chain fatty acids (SCFAs) in feces and serum metabolites were next analyzed. HFD treatment resulted in a marked loss of SCFAs in feces, including acetate, propionate and butyrate, which were fully restored by addition of S or ML inulin (Figure 6a). Oppositely, branched‐chain fatty acids like isobutyrate and isovalerate increased by HFD‐treatment, which was inhibited by S‐ or ML‐inulin addition (Figure 6b).

Further, serum samples from the treated mice with ND, HFD, HFD‐S or HFD‐ML were applied to untargeted metabolomics analysis. Linolenic acid (LA) and alpha‐Linolenic acid (ALA) belonging to omega‐3 and omega‐6 polyunsaturated faddy acids (PUFAs), respectively, are the only two essential fatty acids for humans, who must obtain them from diet. From ALA, human can synthesize two other omega‐3 PUFAs important for cellular functions, including Eicosapentaenoid acid (EPA) and Docosahexaenoic acid (DHA). HFD treatment significantly decreased the serum levels of the above four PUFAs in mice, which were further decreased by addition of S inulin, while addition of ML inulin into HFD did not seem to affect them (Figure 6c). In addition, the levels of Thiamine (Vitamin B1, VB1) and Niacinamide (Vitamin B3, VB3) and Pantothenic acid (Vitamin B5, VB5) were similar between ND and HFD, but all these vitamins were dramatically decreased in mice by HFD treatment compared with mice by ND (Figure 6d and Supplementary Table S4). The supplementation of S and ML inulin in HFD further decreased the levels of VB1 and VB3. Similar reductions in PUFA and vitamin B were also observed in feces, indicating the gut microbiota contribute to it. Compared with ND treatment, HFD treatment substantially decreased the levels of serum carnitine and its derivatives, which were partly restored by addition of ML inulin into HFD (Figure 6e).

## 4 DISCUSSION

The present study clearly compared the effects of S‐inulin (DP: 2–9) and ML‐inulin (DP: 17–24) on HFD‐induced obesity, fatty liver, inflammation and insulin sensitivity. Although previous studies have reported that inulin addition was related to improve metabolic disorders in human and rodents, so many controversial reports made some confusions in its anti‐obesity and insulin sensitivity (Bonsu & Johnson, 2012; Kumar et al., 2016; Zou et al., 2018). The present study showed both S‐inulin and ML‐inulin supplementation significantly increased energy expenditure (Figure 2), alleviated fatty liver (Figure 1c and 1d). Very interestingly, ML‐inulin administration was shown more effective than that of S‐inulin in HFD‐induced obesity, inflammation and insulin sensitivity. One of main contributions in present study was to clarify that ML‐inulin addition alleviated HFD‐induced obesity with solid data. This could be explained by increased fatty acid oxidation and oxidative phosphorylation in adipose tissue. Further direct molecular mechanism is still unknown how inulin administration affected energy expenditure through adipose tissue, thus, this needs further investigation.

The second interesting and novel finding in the present study came from the metabolomics analysis and it demonstrated dramatically HFD‐induced decrease in PUFA and vitamin B family, which cannot be rescued but further reduced by inulin administration (Figure 6). The reduction in PUFA by HFD was consistent with the previous study that HFD‐fed rats had significantly lower level of PUFA in plasma compared with ND‐fed rats (An et al., 2013). Studies have reported that omega‐3 fatty acid deficiency linked to lower intelligence, depression, heart disease, cancer and many other health problems, while lack of omega‐6 fatty acid was associated with severe dermatitis (Fujii et al., 2013; Labrousse et al., 2018; Mathieu et al., 2011). Since vitamin B are necessary micronutrients that synthesized by plants and bacteria, not by mammals, thus, mammals must obtain vitamin B from dietary or microbial sources like the gut microbiota (Yoshii et al., 2019). However, vitamin B producers, including *Bacteroides*, *Prevotella*, *Lactobacillus* and *Helicobacter*, were markedly decreased in the intestine of HFD‐treated mice compared with ND‐treated mice, which probably in part accounted for the reductions in vitamin B in feces and serum (Magnúsdóttir et al., 2015; Yoshii et al., 2019). Not only essential fatty acids, but also vitamin B1 and vitamin B3 are probably needed to make up for the loss caused by intake of S or ML inulin.

Our results revealed that inulin as a prebiotic agent has improved HFD‐induced dysbiosis of gut microbiota to a certain extent. Our study was partially in agreement with the results by Zhao et al. (Zhao et al., 2018) that intake of a fiber‐rich diet for 28 days caused a notable reduction in gut microbiota diversity (gene richness) of patients with type 2 diabetes, which challenged the current notion that greater overall diversity implies better health (Le Chatelier et al., 2013; Lozupone et al., 2012). Also, supplementation with S or ML inulin restored *Firmicutes*/*Bacteroidetes* ratio induced by HFD, which was thought to be negatively associated with both obesity and diabetes (Ley et al., 2005, 2006); it also reduced relative abundance of *Proteobacteria*, which have been suggested to promote an array of chronic inflammatory diseases. At the family level, both S and ML inulin treatment markedly increased the abundance of *Bacteroidetes* *S 24–7*, which is actively involved in the degradation of carbohydrates (plant glycan, host glycan, and α‐glucan), and the increased abundance has been described in mice fed with a low‐fat diet (Ormerod et al., 2016). We observed that both S and ML inulin decreased the relative abundance of endotoxin‐producing bacteria *Desulfovibrionaceae*, which was in line with previous study showing that the relative abundance of *Desulfovibrionaceae* was reduced by the treatment of the probiotic‐enriched diet in obese individuals (Xiao et al., 2014). The relative abundance of *Alistipes* have been reported to be negatively correlated with obesity and associated metabolic disorders, and their abundances were also decreased by S or ML inulin in HFD‐fed mice (Geurts et al., 2011; Zhang et al., 2012). We also found the relative abundances of other endotoxin‐producing bacteria, including *Oscillibacter*, *Ruminiclostridium* and *Desulfovibrio* decreased after S or ML treatment, which was associated with obesity and other associated metabolic disorders (Golubeva et al., 2015; Guo et al., 2018; Kim et al., 2012). *Akkermansia muciniphila* is a highly promising probiotic, and is closely associated with the nutrition metabolism. The growing body of evidence has proposed its great potential for the prevention and treatment of obesity, insulin resistance, adipose tissue inflammation and their associated metabolic disorders in the intestinal tract for rodents (Caesar et al., 2015; Everard et al., 2013; Zhou, 2017). Our result was consistent with one previously published study that feeding of prebiotics to genetically obese mice increased the abundance of *Akkermansia muciniphila* by approximately 100‐fold (Everard et al., 2011).

We have to note different effects between S and ML inulin although some similar tendency was observed in this study, including alleviating fatty liver, increased energy expenditure and even similar gut microbiota. The supplementation of ML inulin has been shown better effects to improve diet‐induced disorders than that of S inulin. More energy expenditure and less inflammation in ML‐inulin treated mice than S‐inulin treated mice were possibly main direct causes. These differences may be due to the differences in key microbiota, such as *Roseburia*, *Akkermansia*, *Dorea*, *Lactobacillus*, which could be rescued more in ML‐inulin treated gut than that of S‐inulin treated gut. The study possibly provided some tips to make probiotic strains combination from these genera for obesity and diabetes therapy.

In conclusion, our study revealed that ML inulin treatment can improve the obesity‐related indexes, chronic inflammation, insulin sensitivity, energy metabolism and composition of gut microbiota in diet‐induced obese mice, indicating ML inulin as a natural alternative for anti‐obesity.

## CONFLICT OF INTEREST

There are no conflicts of interest to declare.

## Supporting information

Fig S1

Table S1

Table S2

Table S3

Table S4
