# Supplementary figures and images for: Chain length‐dependent inulin alleviates diet‐induced obesity and metabolic disorders in mice
Source: Food Sci Nutr. 2021 May 7;9(7):3470–82. doi: 10.1002/fsn3.2283 (PMC8269689; doi:10.1002/fsn3.2283)

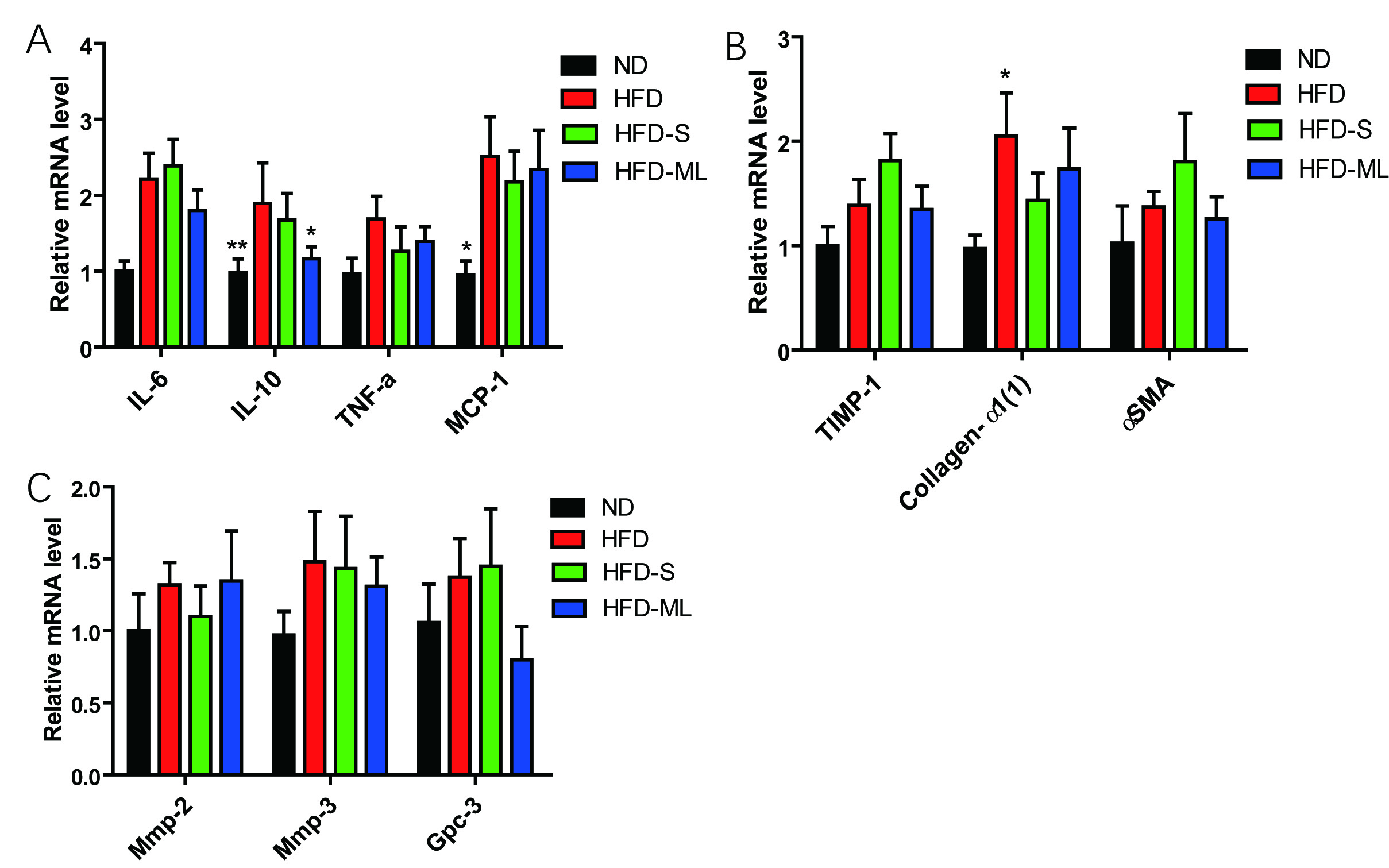

Supplement: Supplementary file 1 — Fig S1 [file FSN3-9-3470-s002.jpg]
