# Supplementary material for: Chain length‐dependent inulin alleviates diet‐induced obesity and metabolic disorders in mice
Source: Food Sci Nutr. 2021 May 7;9(7):3470–82. doi: 10.1002/fsn3.2283 (PMC8269689; doi:10.1002/fsn3.2283)
Supplement: Supplementary file 2 — Table S1 [file FSN3-9-3470-s004.docx]

| **Ingredient** | **Grams** |
| --- | --- |
| Protein | 191 |
| Carbohydrate | 187.8 |
| Fiber | 74 |
| Fat | 261 |
| Mineral | 50 |
| Vitamin | 3 |
| Dye | 0.05 |
| Total | 766.85 |

Supplementary table 1. The major composition of HFD
