# Supplementary material for: Chain length‐dependent inulin alleviates diet‐induced obesity and metabolic disorders in mice
Source: Food Sci Nutr. 2021 May 7;9(7):3470–82. doi: 10.1002/fsn3.2283 (PMC8269689; doi:10.1002/fsn3.2283)
Supplement: Supplementary file 3 — Table S2 [file FSN3-9-3470-s003.docx]

Supplementary table 2. The total calories of HFD, HFD-S and HFD-ML.

| **Diets** | **Calories (Kcal/g)** |
| --- | --- |
| HFD | 5.10 |
| HFD-S | 4.97 |
| HFD-ML | 5.03 |
