# Supplementary material for: Chain length‐dependent inulin alleviates diet‐induced obesity and metabolic disorders in mice
Source: Food Sci Nutr. 2021 May 7;9(7):3470–82. doi: 10.1002/fsn3.2283 (PMC8269689; doi:10.1002/fsn3.2283)
Supplement: Supplementary file 4 — Table S3 [file FSN3-9-3470-s001.docx]

Supplementary table 3. Sequences of primers used for qRT-PCR analysis

|  | Forward (5’-3’) | Reverse (5’-3’) |
| --- | --- | --- |
| Actin | GGCTGTATTCCCCTCCATCG | CCAGTTGGTAACAATGCCATGT |
| ACC1 | AGCTGATCCTGCGAACCT | GCCAAGCGGATGTAAACT |
| FAS | TCCAAGACTGACTCGGCTACTGAC | GCAGCCAGGTTCGGAATGCTATC |
| SCD1 | TTCTTGCGATACACTCTGGTGC | CGGGATTGAATGTTCTTGTCGT |
| DGAT1 | TCCGTCCAGGGTGGTAGTG | TGAACAAAGAATCTTGCAGACGA |
| DGAT2 | CTGGCTGATAGCTGCTCTCTACTTC | TGTGATCTCCTGCCACCTTTC |
| SREBP1c | GGAGCCATGGATTGCACATT | GCTTCCAGAGAGGAGGCCAG |
| ATGL | GGAGACCAAGTGGAACATCTCA | AATAATGTTGGCACCTGCTTCA |
| PPARα | ACGGCAATGGCTTTATCA | CGCTGCGTCGGACTCGGT |
| CPT1 | ACCACTGGCCGCATGT | CTCCATGGCGTAGTAGTTGCT |
| CPT2 | CAGCACAGCATCGTACCCA | TCCCAATGCCGTTCTCAAAAT |
| Cox4 | CGGCGTGACTACCCCTTG | TGAGGGATGGGGCCATACA |
| Cyto C | CCAAATCTCCACGGTCTGTTC | ATCAGGGTATCCTCTCCCCAG |
| Acadl | GCATCAACATCGCAGAGAAA | ACGCTTGCTCTTCCCAAGTA |
| Acadm | GCTAGTGGAGCACCAAGGAG | CCAGGCTGCTCTCTGGTAAC |
| TNFα | CCAGACCCTCACACTCAGATC | CACTTGGTGGTTTGCTACGAC |
| IL-6 | CCAGAGATACAAAGAAATGATGG | ACTCCAGAAGACCAGAGGAAAT |
| IL-18 | GACTCTTGCGTCAACTTCAAGG | CAGGCTGTCTTTTGTCAACGA |
| MCP1 | AGGTCCCTGTCATGCTTCTG | GCTGCTGGTGATCCTCTTGT |
| UCP1 | ACTGCCACACCTCCAGTCATT | CTTTGCCTCACTCAGGATTGG |
| Cidea | TCCTCGGCTGTCTCAATG | TGGCTGCTCTTCTGTATCG |
| TIMP-1 | GCAACTCGGACCTGGTCATAA | CGGCCCGTGATGAGAAACT |
| Collagen-α1(1) | GCTCCTCTTAGGGGCCACT | CCACGTCTCACCATTGGGG |
| αSMA | GTCCCAGACATCAGGGAGTAA | TCGGATACTTCAGCGTCAGGA |
| MMP-2 | CAAGTTCCCCGGCGATGTC | TTCTGGTCAAGGTCACCTGTC |
| MMP-3 | ACATGGAGACTTTGTCCCTTTTG | TTGGCTGAGTGGTAGAGTCCC |
| Gpc-3 | CAGCCCGGACTCAAATGGG | CAGCCGTGCTGTTAGTTGGTA |
