# Supplementary material for: Chain length‐dependent inulin alleviates diet‐induced obesity and metabolic disorders in mice
Source: Food Sci Nutr. 2021 May 7;9(7):3470–82. doi: 10.1002/fsn3.2283 (PMC8269689; doi:10.1002/fsn3.2283)
Supplement: Supplementary file 5 — Table S4 [file FSN3-9-3470-s005.docx]

| **Vitamins** | **ND** | **HFD** |
| --- | --- | --- |
| VB1 (μg/g) | 61.2 | 55.5 |
| VB5 (μg/g) | 69.7 | 60.4 |
| VB6 (μg/g) | 28.8 | 24.4 |

Supplementary table 4. The composition of vitamins in ND and HFD
